# Supplementary figures and images for: The impact of primary colonizers on the community composition of river biofilm
Source: PLoS One. 2023 Nov 13;18(11):e0288040. doi: 10.1371/journal.pone.0288040 (PMC10642824; doi:10.1371/journal.pone.0288040)

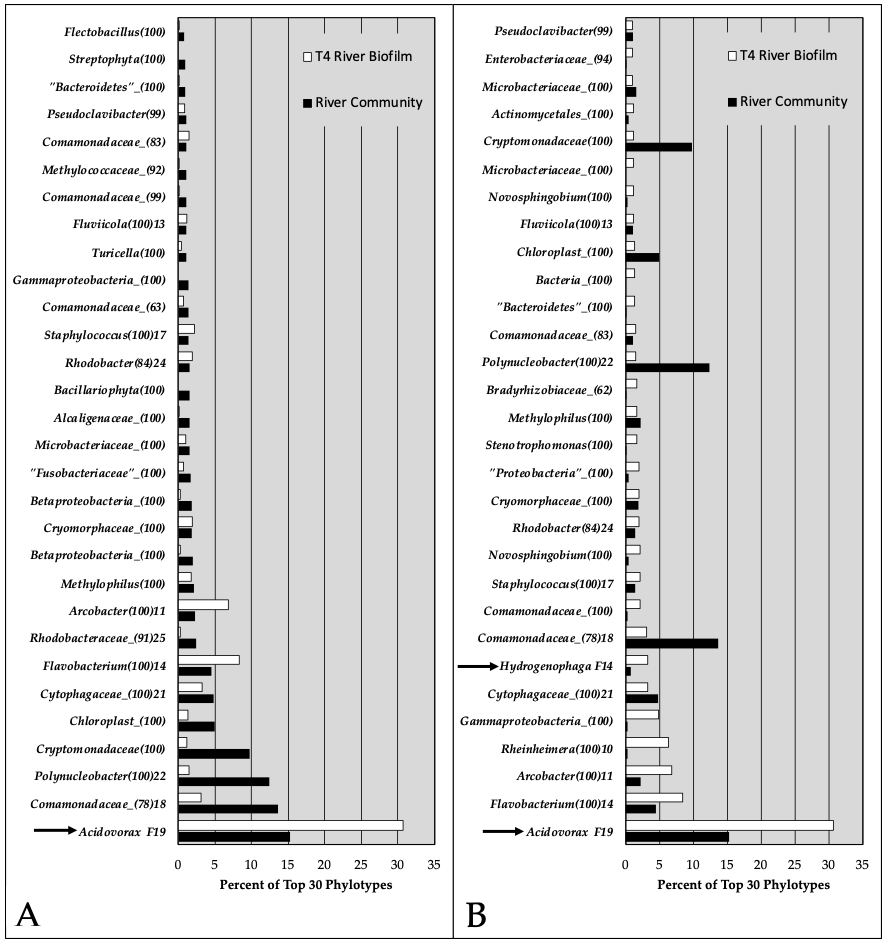

Supplement: S1 Fig — Panels A, C & E are sorted according to the numerical abundance of river water populations. Panels B, D & F are sorted according to the numerical abundance of river biofilm populations. Panels A&B, C&D and E&F are samples at 4, 8 and 24 hours respectively. Populations that were isolated from eggs in previous studies are indicated with arrows. (ZIP) [file pone.0288040.s001.zip › S1AB_Fig.tif]

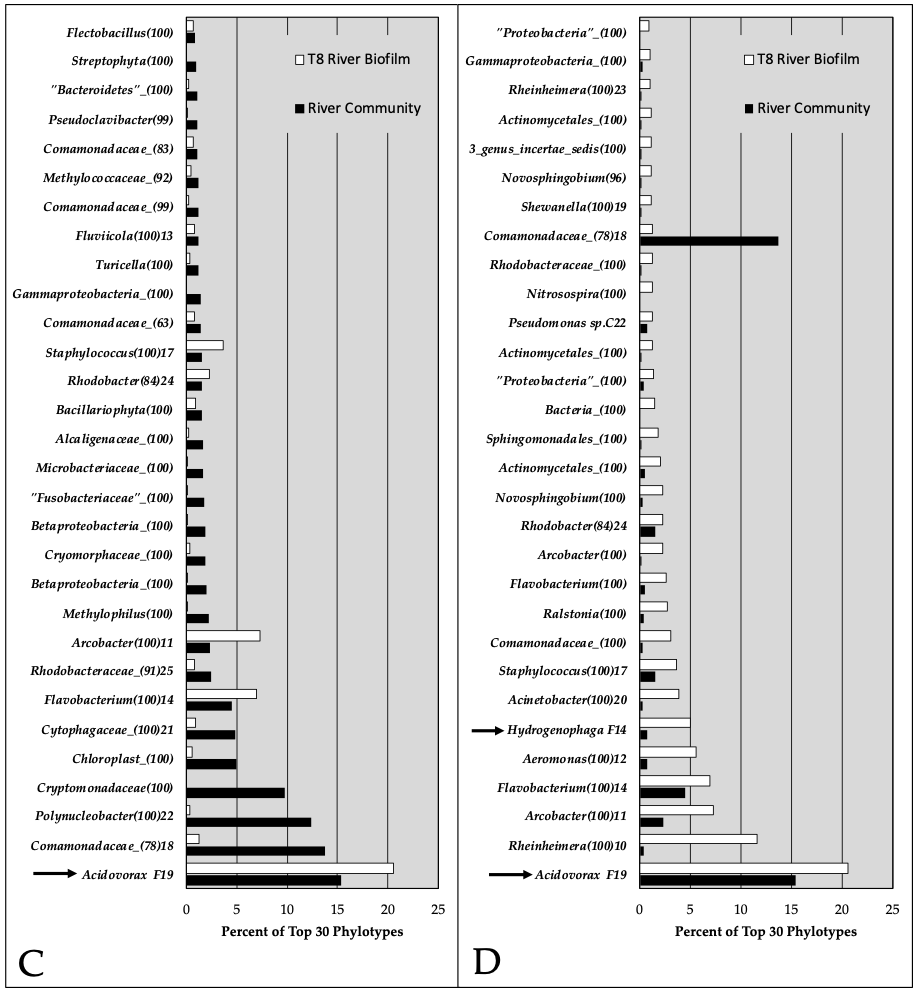

Supplement: S1 Fig — Panels A, C & E are sorted according to the numerical abundance of river water populations. Panels B, D & F are sorted according to the numerical abundance of river biofilm populations. Panels A&B, C&D and E&F are samples at 4, 8 and 24 hours respectively. Populations that were isolated from eggs in previous studies are indicated with arrows. (ZIP) [file pone.0288040.s001.zip › S1CD_Fig.tif]

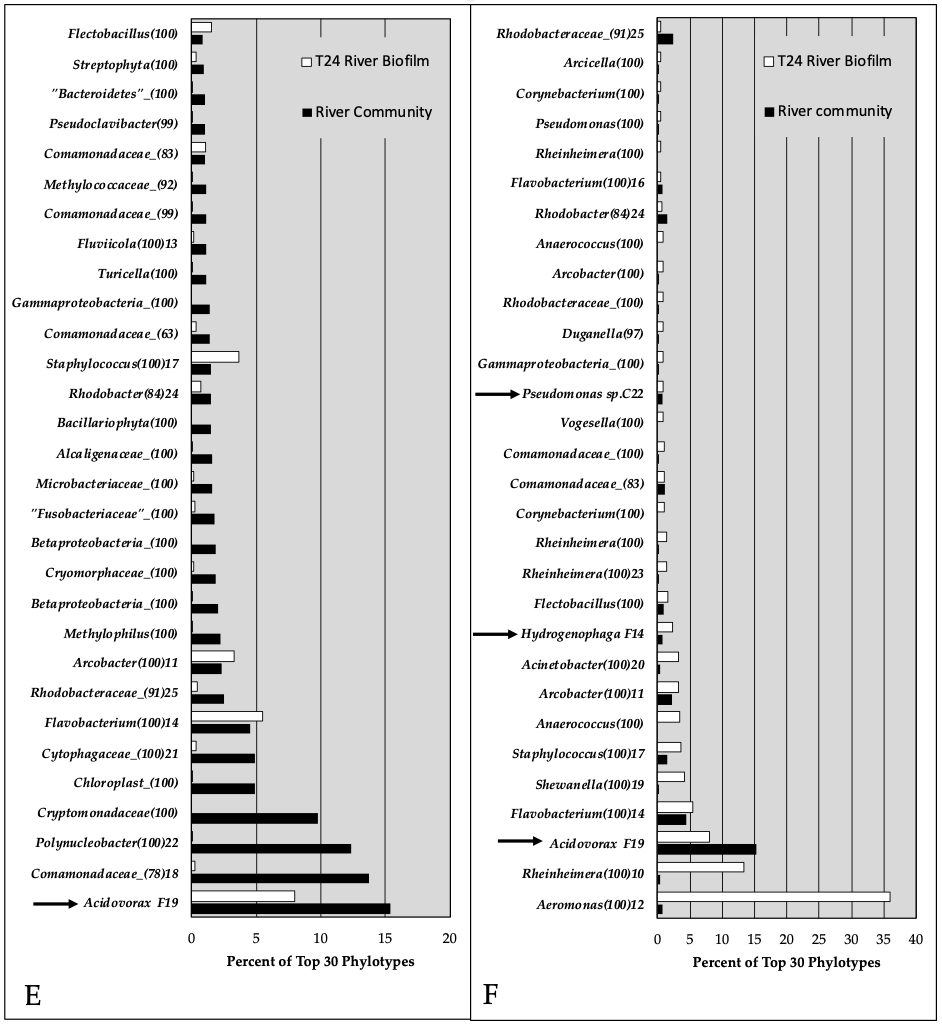

Supplement: S1 Fig — Panels A, C & E are sorted according to the numerical abundance of river water populations. Panels B, D & F are sorted according to the numerical abundance of river biofilm populations. Panels A&B, C&D and E&F are samples at 4, 8 and 24 hours respectively. Populations that were isolated from eggs in previous studies are indicated with arrows. (ZIP) [file pone.0288040.s001.zip › S1EF_Fig.tif]

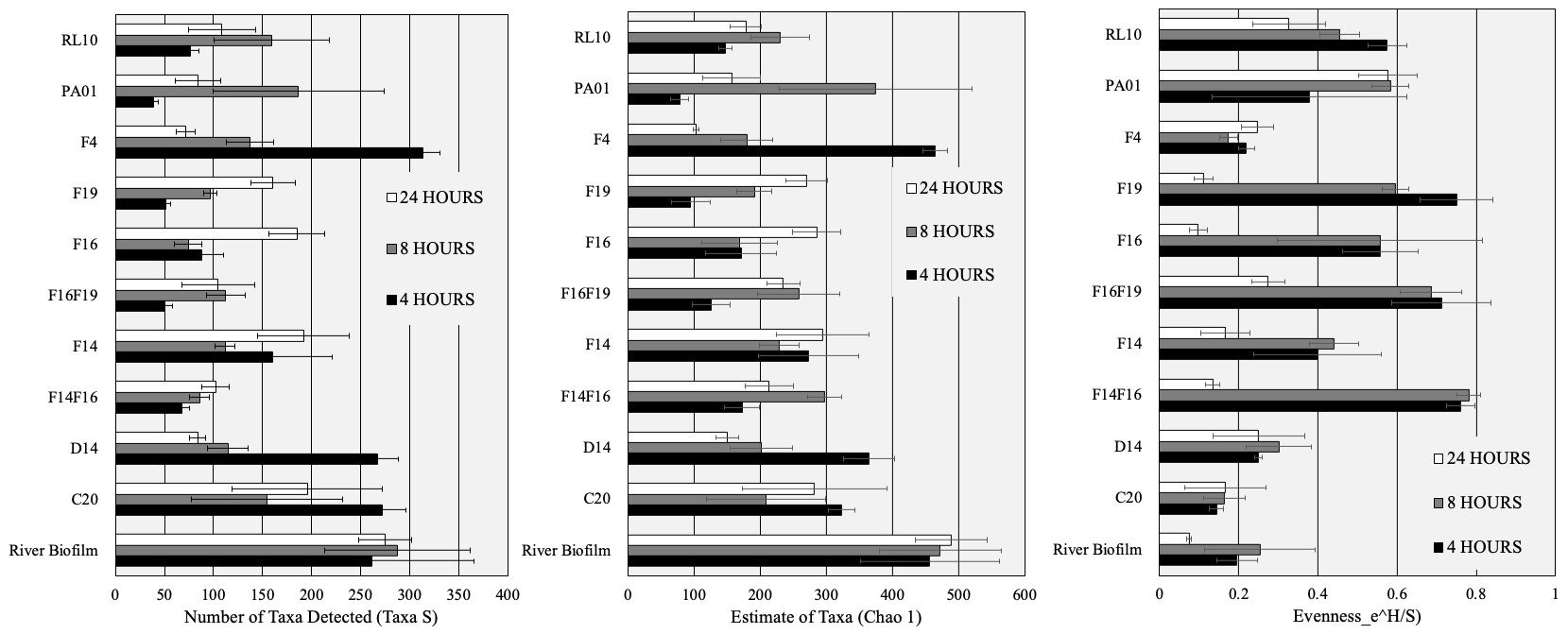

Supplement: S2 Fig — These measurements are derived from community analyses from which the founding isolate populations have been subtracted, thereby providing values that are not substantially influenced by the large population size of the established biofilms. Four replicates were used to calculate the mean and standard deviation. (TIF) [file pone.0288040.s002.tif]

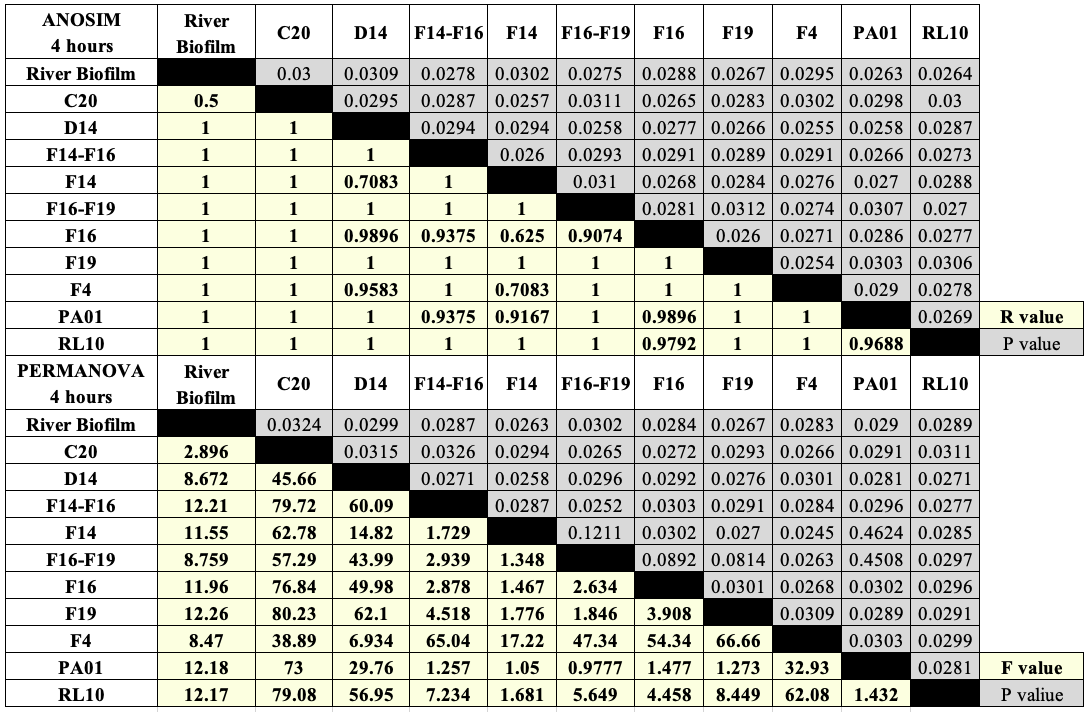

Supplement: S3 Fig — S3A, 4hrs, S3B, 8hrs, S3C, 24hrs. (ZIP) [file pone.0288040.s003.zip › S3A_Fig.tif]

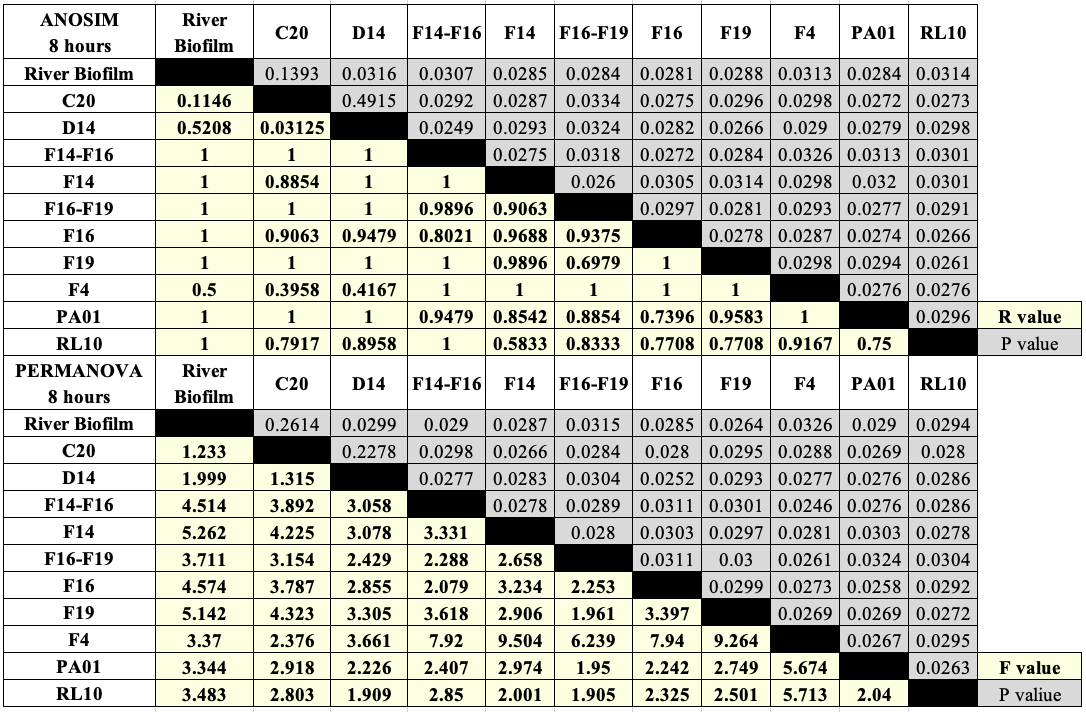

Supplement: S3 Fig — S3A, 4hrs, S3B, 8hrs, S3C, 24hrs. (ZIP) [file pone.0288040.s003.zip › S3B_Fig.tif]

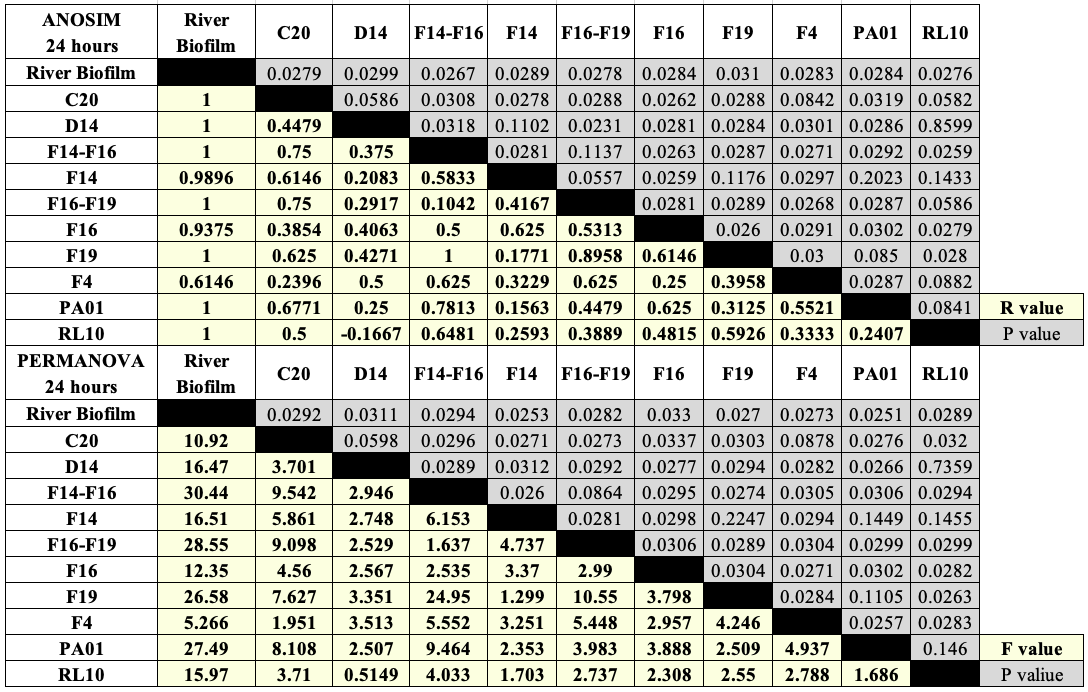

Supplement: S3 Fig — S3A, 4hrs, S3B, 8hrs, S3C, 24hrs. (ZIP) [file pone.0288040.s003.zip › S3C_Fig.tif]

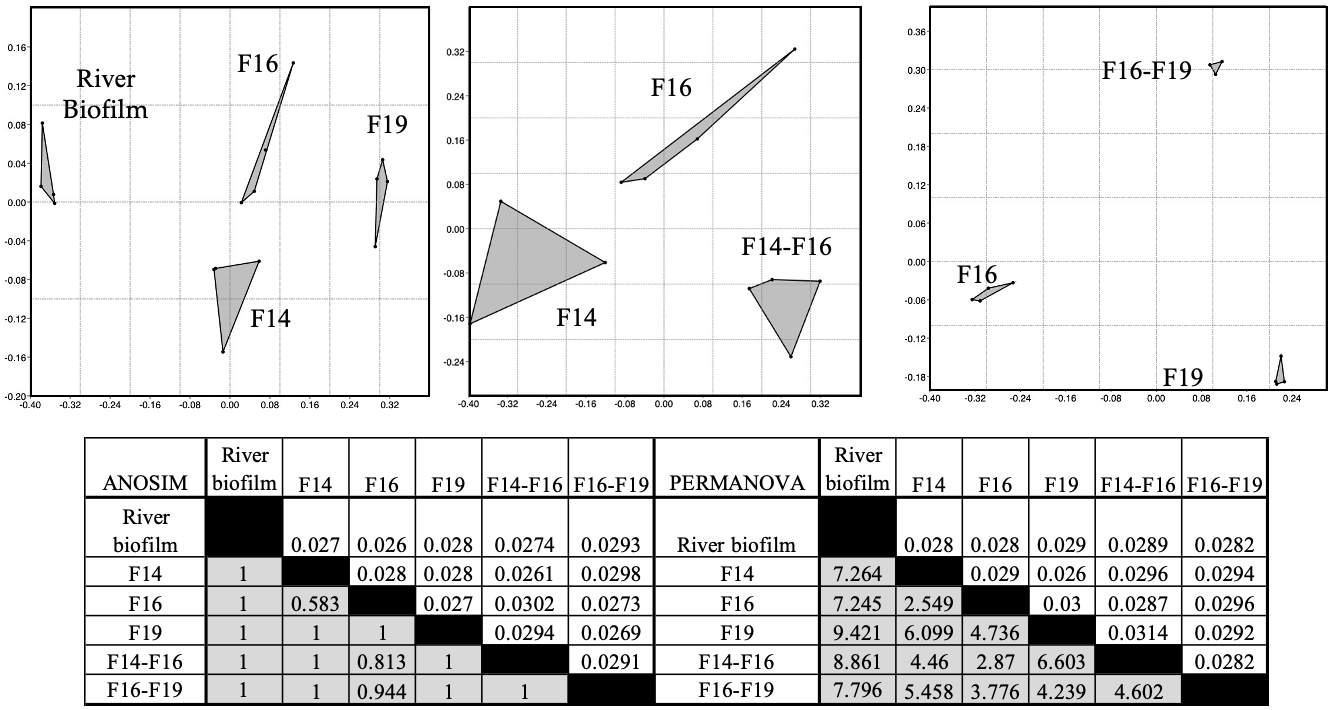

Supplement: S4 Fig — Founding populations of the pre-established communities have been removed prior to comparative analyses. R values and F values are shaded, and uncorrected P values are unshaded. (TIF) [file pone.0288040.s004.tif]

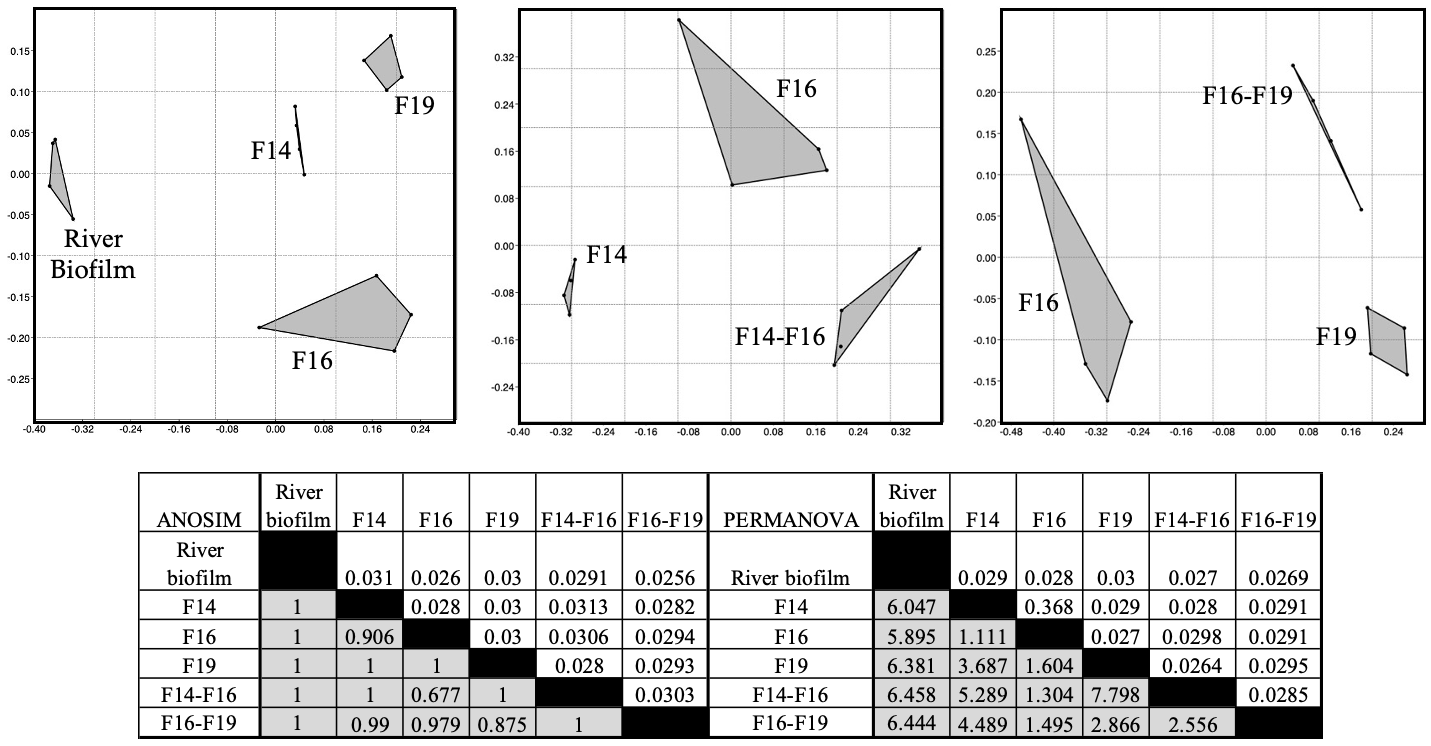

Supplement: S5 Fig — Founding populations of the pre-established communities have been removed prior to comparative analyses. R values and F values are shaded, and uncorrected P values are unshaded. (TIF) [file pone.0288040.s005.tif]
