# Supplementary material for: The impact of primary colonizers on the community composition of river biofilm
Source: PLoS One. 2023 Nov 13;18(11):e0288040. doi: 10.1371/journal.pone.0288040 (PMC10642824; doi:10.1371/journal.pone.0288040)
Supplement: Attachment — Submitted filename: Response to Reviewers.doc [file pone.0288040.s006.doc]

November 30, 2026

PLOS
1265 Battery Street, Suite 200
San Francisco, CA 94111
United States

RE: PONE-D-22-31146

The impact of primary colonizers on the community composition of river biofilm

Colleagues at PLOS ONE,

We are submitting a revised manuscript to PLOS ONE titled “**The impact of primary colonizers on the community composition of river biofilm”.** Our thanks to the editors and reviewers who have helped to improve the paper.

Our response to the reviewers is below. Bold text are comments from the reviewers and highlighted text contains our response.

Best regards,

Terence L. Marsh, Ph.D.

Assoc. Professor Emeritus

Dept. of Microbiology & Molecular Genetics

567 Wilson Road

Michigan State University

East Lansing, MI 48824

(517)-231-6033

MARSHT@msu.edu

PONE-D-22-31146

The impact of primary colonizers on the community composition of river biofilm

Response to Reviewers

When submitting your revision, we need you to address these additional requirements.

1. Please ensure that your manuscript meets PLOS ONE's style requirements, including those for file naming. The PLOS ONE style templates can be found at 
We have check this including file names.

2. Thank you for stating the following in the Acknowledgments Section of your manuscript: 
   "Funding sources included the Michigan Department of Natural Resources State Wildlife Grants Program T-10-T-5 Study 237026 (<https://www.michigan.gov/dnr/buy-and-apply/grants/aq-wl/wildlife562> hab), Center for Water Sciences Water Cube initiative at MSU (<https://water.msu.edu/watercube/>), and the Department of Microbiology and Molecular Genetics at MSU. In addition, we thank the Department of Microbiology and the College of Natural Science for partial funding including a Thesis Completion grant. We thank Dr. M. Bagdasarian (MSU) for providing Pseudomonas aeruginosa PA01."

We note that you have provided funding information that is not currently declared in your Funding Statement. However, funding information should not appear in the Acknowledgments section or other areas of your manuscript. We will only publish funding information present in the Funding Statement section of the online submission form. 
**Please remove any funding-related text from the manuscript and let us know how you would like to update your Funding Statement. Currently, your Funding Statement reads as follows:**

DONE

  "Funding sources included the Michigan Department of Natural Resources State Wildlife Grants Program T-10-T-5 Study 237026 to KS (<https://www.michigan.gov/dnr/buy-and-apply/grants/aq-wl/wildlife-hab>), Center for Water Sciences Water Cube initiative at MSU to TLM & KS (<https://water.msu.edu/watercube/>), and the Department of Microbiology and Molecular Genetics at MSU (TLM). In addition, we thank the Department of Microbiology and the College of Natural Science for partial funding including a Thesis Completion grant (RA). The funders had no role in study design. data collection and analysis, decision to publish, or preparation of the manuscript."

**Please include your amended statements within your cover letter; we will change the online submission form on your behalf.**

The current funding statement is adequate.

Reviewers' comments:
**Comments to the Author**

Reviewer #1: General

**The authors investigated how the establishment of biofilms from river microbial communities were affected by pre-established biofilms by isolates from sturgeon eggs and vice versa. They conclude that certain taxa could act as probiotics if they are precolonized on sturgeon eggs and then exposed to possibly harmful taxa in the river communities. In general, I think this is a straightforward and interesting study, however there are several improvements that could be made to the writing and presentation of these data, and important methodology is missing.**

**The justification for using specific isolates should be highlighted more clearly.**

Additional information has been added.

*The isolates were representative of the culturable phylogenetic diversity found on the egg. In nature, the bacterial communities on eggs are diverse with hundreds of detectable operational taxonomic units. In addition, these isolates were of particular interest because of previous characterizations of biofilm formation and resilience (42, 43). All isolates were known to produce biofilm at levels that were strain-dependent under our test conditions (42). Preliminary work with Acidovorax colonization of freshly fertilized sturgeon eggs suggested that egg survival in a hatchery setting was improved (26). Bacillus C20 and Serratia D14 inhibited growth of Flavobacterium sp. C05, F. columnare 090702-1 and Aeromonas salmonicida 05100658A59, known fish pathogens, in agar overlay tests (42). Furthermore, we knew that established biofilms of Acidovorax F19, Brevundimonas F16 and Hydrogenophaga F14 were disrupted by a Pseudomonas strain commonly found in freshwater, including the Red Cedar River. Serratia D14 was particularly aggressive in soft agar overlay tests against four of the 25 tested isolates from sturgeon eggs, including Hydrogenophaga F14, which was among the most sensitive in these tests (42). In addition, combinations of Brevundimonas and Acidovorax or Brevundimonas and Hydrogenophaga produced more biofilm when paired than single species biofilms (43). Finally, single species biofilms of these three strains had reduced metabolic activity when challenged with tobramycin but the mixed species biofilm of Acidovorax and Brevundimonas had increased metabolic activity when tobramycin was present (43). These attributes of the seven isolates from sturgeon eggs led to their selection for this study.*

**I do not see any informative on cell counts or growth phase at which these isolates were inoculated, and I’m not sure the absorbance values presented would allow for successful replication of these results**.

We have added additional information regarding cultivation of the strains and establishment of biofilm. However, this is something that we considered at the outset of the experiments. I will emphasize at this point that the important element of the study is the formation and resilience of biofilm and that the biofilm biomass produced over 48 hours is species-dependent. The cell concentration of the inoculum is irrelevant to the amount of biofilm formed in 48 hours over a wide range of initial cell concentrations. We have determined this in studies with 500-600 freshwater isolates, including those that we used in this study. Of course, this is assuming that the cells are healthy. We took precautions to assure that we were using only healthy cultures. These cultures were inoculated from a single colony and grown for 16 hours at 25°C on a tube roller. The M&M has been updated to emphasize this. It would have been extraordinary if we could have regulated biofilm growth such that each strain or strain combination had the same number of cells per cm^2^ in the biofilm at the start of the challenge. This was clearly beyond our time frame for the experiment. Moreover, as our results indicate, considerably valuable insight can be gained by simply using the biofilm amounts that each strain can produce under our experimental conditions. Far more important to us than equivalent OD at the time of inoculum was the relative crudeness of the crystal violet assay. This assay has a number of pitfalls that can produce variable results and hence large standard deviations. These include the formation of a biofilm pellicle and the loss of biofilm due to tearing during washing and staining. The use of the 24-well microtiter plates rather than the 96 well plates greatly reduced these problems and gave us improved reproducibility. With these plates and an experienced hand our standard deviations were small.

**There are other pieces of information missing from the methods section that I’ve highlighted below that need to be included.

The English is fine, but the scientific writing, especially in the results, could be improved.**

**The results could be tightened by removing statements that are not informative to the results. For example, Lines 265-266 don’t seem to add much and may be more appropriate for a legend, and Lines 284-288 are redundant of intro and methods. There is much more than can be cut.**

**The data presented in the results section and the figures/tables are not always clear. For example Table 1 and Figure 1 panel B and others like it. The legends need to be more way informative so the reader can understand them as well, but in general I think the presentation of data in some tables and figures should be re-considered (Table 1, Figure 1c, Figure 4 [way too small, no description of colors?], Figure 6, Figure 7 [too small of text]). In addition I think the results needs to be described more clearly with more informative information presented in the figures. I could not understand the results without spending time trying to figure out what the figures were presenting, and even then, the legends were missing necessary information.**

Table 1 has been simplified and made larger. See response to comment below.

The legend for Figure 2 has been modified.

***Fig 2. Resilience of single and double isolate biofilms to filtered and unfiltered river water as measured with crystal violet.*** *The plotted 600 nm absorbance values (abscissa) are the average of four replicate biofilms of each of the tested strains (ordinate) and standard deviations are indicated with bars. Striped and gray bars represent biofilm challenged with filtered and unfiltered river water, respectively. Asterisks indicate responses that were statistically different (P value = 0.05 or less) from the cognate control 48-hour biofilm (black bars).*

Figure 3 has been revised with a revised Legend.

***Fig 3. Resilience of Established Biofilm to Challenge with River Bacterial Populations as Determined by 16S Amplicon Sequencing.*** *Panel A, percent (average of four replicates) of the founding populations remaining in biofilms at 4, 8 and 24 hours. Panels B (F14 & F16 mixed biofilm) and C (F19 & F16 mixed biofilm), percent (average of four replicates) of the founding two-isolate biofilms remaining after 4, 8 and 24 hours. Percent is calculated based on the entire community (11,100 sequences), including isolates. Error bars display the standard deviation based on four replicates.*

Figure 4 has been recast to enlarge and a more descriptive legend has been added.

***Fig 4. Cluster Analysis of Biofilms Communities at 4, 8 and 24 hours (T4, T8, T24).*** *Cluster analyses (UPGMA-Bray-Curtis) were performed with (upper row) and without (lower row) the founding populations. Clusters of founding populations are color-coded systematically in all dendrograms to facilitate tracking.*

**I think the conclusion could be more strongly supported if there was a known egg pathogen to challenge the biofilm rather than inferring certain pathogens from the microbial community composition of the river. Certainly not all Aeromonas sp. are pathogenic to the eggs? However, I see the value in investigating as a microbial community as well because it is more representative of nature conditions.**

We debated whether or not to include a known fish pathogen. We feel that this is a good direction to move in but as many of us know, experimental design can amplify up and become unwieldy. When we considered pathogens, within a few minutes we had added an additional 5 pathogenic strains that included *F. columnare*, *F. psychrophilum, A. salmonicida, A. hydrophila*, and a *Yersia sp*. This increased our experimental design by 50 new combinations and 200 additional time points replicated x4, and more sequencing than we could afford. Nonetheless, we feel that our current report helps to push the studies of biofilm assembly and rapid sorting of positive and negative interactions between bacterial populations at the level of biofilm.

Specific
**Line 55 awkward sentence**

Changed to “The aseptic eggs of many aquatic species are expelled into an aquatic environment and are rapidly colonized by microbial populations.”

**Line 82: bd is not defined before this, I assume it is B. dendrobatidis**

Bd changed to *B*. *dendrobatidis*

**Line 83: suggestion: remove “in our labs”**

Removed

**Line 86: suggestion: alter language to avoid first person**

Corrected

**Line 93: I think “with” is missing**

Corrected

**Line 98. Starting with “our experimental strategy…” – consider removing this and everything after or shorten it.**

156 words were reduced to 88. We felt compelled to state in broad strokes the results of our study at the end of the intro.

**The details of how isolates were identified need to be added. For example, you just say that partial 16S sequencing was used to identify.**

We state that partial rRNA sequence was initially used for identification followed by complete rRNA sequence (~1100 bases) as well as genome sequence. The genome sequencing allowed us to use Mash/MinHash to make genome-based comparisons with the database. This is stated in the M&M.

*Isolates from the sturgeon egg were initially identified with partial (300-400 bases) 16S rRNA sequencing (42) and later with nearly complete 16S rRNA sequence (28).*

**In addition, the justification for using these strains is lacking.**

The section has been expanded to better explain the logic of using these isolates. See above.

**For example, line124-125 states that these were representative of previous characterizations – is this something you can cite?**

Yes. Cited above.

**How abundant are these taxa on the eggs? Are egg communities diverse or do they only harbor a few species? Following that at line 126, do you mean that these were of interest related to biofilms and resilience on eggs or just in general?**

All of these questions are covered in the expanded M&M.

**Llines 153 and on.. Can you provide more quantitative information about the overnight cultures inoculated? Cell count? Absorbance? Were they added at the same concentration?**

This issue is addressed above. Briefly, what is important here is the biofilm formed after 48 hours. Healthy 16-hour cultures were used as the inoculum. Each strain has its own unique growth rate and ability to form biofilm. The biomass of biofilm produced by each strain was strain-dependent. Under our experimental conditions the biofilm amounts as measured by crystal violet were consistent for each strain at 48 hours, over a broad range of inoculum concentration. We sought a rapid way to consistently generate biofilm from our isolates.

The following was added to the Biofilm formation section of M&M.

*Biofilm screening assays of over 600 freshwater isolates indicated that the amount of biofilm produced under our experimental conditions (R2broth, 25°C shaking in microtiter plates) was strictly strain-dependent (D. Ye, T.L. Marsh, in preparation). Indicative of this observation was the diversity of biofilm biomass produced by the seven strains included in this study. Each strain produced a unique amount of biofilm in 48 hours as measured by a traditional crystal violet assay and this 48-hour biofilm was what was used in the challenge experiments. In addition, the larger well format afforded by the 24 well plates eliminated concerns regarding air-liquid surface films and damage to biofilms during washing and staining. In previous studies, air-liquid surface biofilms were problematic when the amount of biofilm production was optimized in select strains by the addition of exogenous protein (44). Only solid surface-liquid biofilms were observed under our experimental conditions described here* and no ripping or tearing of biofilm was observed*.*

**Line 160 – avoid starting a sentence with a number.**

Changed.

**Line 204 is awkward. Consider changing to “…500 cycle v2 reagent kit to generated 250 bp paired end reads.**
Changed.

**Line 205 needs work to clarify.**

Changed for clarity.

**Line 212-214 is awkward the way it is written. For example, the analysis of diversity of bacterial communities is redundant to all the following analyses listed.**

Perhaps we have a misunderstanding here. All of these measurements are dependent on the taxonomic identification of sequences from the amplicon library. The analysis of ecological diversity indices uses several classic measures of diversity provided in PAST (eg. Simpson vs Shannon), cluster analysis is of course dependent on the Bray-Curtis dissimilarity matrix derived from the taxonomic analysis of each sample, as is NMDS. ANOSIM and PERMANOVA are dependent on the amplitude and number of OTUs of each sample. We have added “The calculation of ecological diversity indices of bacterial communities…” to clarify what we mean by diversity.

**Line 212: permanova**

Corrected.

**Can you provide the details of your mothur analysis? Trimming, filtering, chimera checks?**
The following was added to the M&M.

*In Mothur, the make.contig command aligns sequence pairs and identifies any mismatched sites and these sites are resolved using the quality scores. For example, where one strand has a gap and the other a base, the base is considered valid only when the quality score of the base is above 25. If there are two different bases at a position, then the base with a quality score six greater or more is taken as the base call. If the quality score difference is less than six the consensus base is set to N. The resulting contigs are then screened and all sequences with ambiguous bases or longer than the expected length are removed. The resulting collection of sequences are trimmed to similar lengths. Mothur uses the chimera.vsearch command to identify and remove chimeras. This program is in the public domain and available at Github.*

**Isn’t is “t-test?”**

Corrected

**Lines 228: “were” instead of was**

Corrected

**The first paragraph of the results section doesn’t seem appropriate for the results, but perhaps intro or beginning of discussion.**

Most of this paragraph has been removed.

**Line 238: Is there data somewhere to show this or reference?**

Removed sentence. Part of Results section reduction.

**Is the sentence starting at line 239 needed? Also seems like the next two sentences are redundant of the methods.**

Removed. Part of Results section reduction

**Line 245: I don’t think stress is in an indicator of anything being different but rather an indicator of how well the data is represented in that 2 or 3D model.**

Changed to; *“The stress values for 2 and 3 dimensional plots are 0.1081 and 0.0683, respectively, indicating good to excellent ordination with little or no chance of misinterpretation.*

**Panel B in Figure 1 is not easy to interpret. The shaded boxes and non-shaded boxes need to be explained in the legend. According to the way it is now, one could interpret that T4, T8 etc are R values, because it is not clear here these are timepoints.

In figure 1 panel C what does Taxa S mean? Describe these things in the legend.**

Figure 1 has been corrected and the figure legend has been expanded to hopefully clear up any possible misunderstanding of time points and R or F values.

***Fig 1. Ordination, statistical analyses and diversity measures of the river and river biofilm communities.*** *A. Nonmetric multidimensional scaling analysis of replicates from river and river biofilms at 4, 8 and 24 hours (T4, T8 and T24). B. Pairwise ANOSIM (left block) and PERMANOVA (right block) analyses of the river and biofilm communities at 4, 8 and 24 hours (T4, T8 and T24). Note that the R and F values are black text on white background and corresponding P values are white text on black background. C. Diversity measures of river and biofilm communities. Diversity measurements were calculated from the sum of 4 replicates for each sample. Taxa_S is the total number of taxa detected, Simpson 1_D is the inverse of the standard Simson diversity measure and Shannon H is the Shannon-Wiener index.*

**Line 267, what are 164 and 719? Rank abundance? You mention relative abundance right before this so this is a bit confusing.**

Tried to clarify this. We have changed to the following.

*For example, the archetypal freshwater populations of Polynucleobacter and Cryptomonadaceae, third and fourth in rank abundance in river water, dropped to 164 and 719 rank abundance, respectively, in 24-hour biofilm. Panel B presents the top 25 phylotypes in the 24-hour biofilm. The column to the right indicates the rank abundance of these populations in river water.*

Thus, we are comparing the rank abundance of river water populations to the same populations but in the rank abundance profile of 24-hour biofilm.

**Table one is not clear as is. The lettering is confusing and not intuitive.**

We have reduced the amount of data in Table 1 to make it easier to read and hopefully clearer. There is still much information in the table, and it does require some study. Unfortunately, we don’t quite know what the reviewer meant by “The lettering is confusing and not intuitive”. We have followed a traditional table format with a title on top and salient points within identified by small bold letters in parentheses that are then explained below the table. The accompanying text in the manuscript has been changed as follows.

*In Panel A of* ***Table 1*** *the top 25 most abundant phylotypes detected in Red Cedar River water are shown. To highlight the shifts in community structures, the columns labeled 4, 8 and 24 hours indicate the rank abundance of these river phylotypes in the developing biofilm. Twelve of the top 25 river populations were among the top 25 populations found in biofilm at four hours. This dropped to six at eight hours and finally five in the 24-hour biofilm. Most river populations in the top 25 experienced a rapid drop in relative abundance in the developing biofilm. For example, the archetypal freshwater populations of Polynucleobacter and Cryptomonadaceae, third and fourth in rank abundance in river water, dropped to 164 and 719, respectively, in 24-hour biofilm. Panel B presents the top 25 phylotypes in the 24-hour biofilm. The column to the right indicates the rank abundance of these populations in river water. The five persistent river populations from the top 25 that were retained in the 24-hour biofilm are indicated by the white arrows and include Acidovorax F19, Flavobacterium sp., Arcobacter sp., Rhodobacter sp. and Staphylococcus sp. Only three populations of the top 25 in the 24-hour biofilm (Flavobacterium OUT00014, Staphlococcus OTU00017 and Hydrogenophaga OTU00003) had positive correlations with their population sizes in river water. Three populations of 24-hour biofilm, identified as Acidovorax F19, Hydrogenophaga F14 and Pseudomonas C22 (shaded cells) were previously isolated from sturgeon eggs (27). Noteworthy is the observation that some populations that appeared in the top 25 of the 24-hour biofilm were extraordinarily low in rank abundance in river water, rising from a ranking of 2000+ in some cases. The top 25 populations of river biofilm at 4-, 8- and 24-hours in comparison with the river community is shown is Supplemental Fig 1. The Taxa_S, Chao 1 and Evenness measures are presented in Supplemental Fig 2.*

**Line 320 “described”**

Corrected.
 **Lines 297 : an absorbance of 3.7 means nothing without any comparisons, which do not follow. What were the absorbances of the “low amounts” of biofilm? What is considered “low” and why?**

**Changed to:**

*Under our experimental conditions (R2broth at 25°C), P. aeruginosa PA01 produced a robust signal of ~3.7 A_600nm_ at 48 hours in the crystal violet assay, indicating considerable biomass in the biofilm that traps crystal violet. Compare this value with considerably weaker biofilms produced by Serratia spp, Hydrogenophaga and Acidovorax that are 1/10 the value.* **The discussion could be streamlined and made me succinct.**

We have tried. This simple experiment generated a considerable amount of data and even though the discussion seems long, there were parts of the data on which we did not comment.

Reviewer #2: Comments from the Reviewer of the Angoshtari et al. manuscript ‘The impact of primary colonizers on the community composition of river biofilm’ (PLoS ONE)

Angoshtari et al. present their research focussed on understanding how the first bacterial colonisers impact the subsequent recruitment and growth of other bacteria present in river water with the aim of better understanding how pathogens might be prevented from colonising sturgeon eggs and causing disease.
This is a very well-written manuscript and I have no serious concerns with it, other than to say it might be edited for length in paces, and that the print-quality of the figures was poor.

Comments

**1. P. aeruginosa PA01 is not necessarily thought of as a model for river bacteria, and it should be made clear when it is first referred to (after the Methods) that P. aeruginosa strains are regularly recovered from fresh water systems.**

Done

*Pseudomonas aeruginosa PAO1 was the generous gift of Dr. Michael Bagdasarian at Michigan State University. This strain was used as a strong positive control for biofilm formation and is routinely isolated from freshwater sources (44, 45, 46, 47) including the Red Cedar River (A. Chan & T.L. Marsh, unpublished), the source of river water for these studies.*

**2. Pg. 5, line 114. The species of sturgeon should be provided.**

Done

*Acipenser fulvescens*
 **3. Pg. 6, line 127 and elsewhere. Whenever percentages are given, w/w, w/v or v/v should be stipulated in parentheses.**

Done

L145, L162, L204, L207, L221
 **4. Pg. 8, line 175. It should be stated clearly that the type of biofilm being examined and measured in this work is a submerged solid surface – liquid (A-L) interface biofilm. Other types of biofilm are equally well-studies, including air – liquid surface interface biofilms that readily form in static microtitre dish assays.**

The following was added to M&M.

*Biofilm screening assays of over 600 freshwater isolates indicated that the amount of biofilm produced under our experimental conditions (R2broth, 25°C shaking in microtiter plates) was strictly strain-dependent (D. Ye, T.L. Marsh, in preparation). Indicative of this observation was the diversity of biofilm biomass produced by the seven strains included in this study. Each strain produced a unique amount of biofilm in 48 hours as measured by a traditional crystal violet assay and this 48-hour biofilm was what was used in the challenge experiments. In addition, the larger well format afforded by the 24 well plates eliminated concerns regarding air-liquid surface films. In previous studies, air-liquid surface biofilms were problematic when the amount of biofilm production was optimized in select strains by the addition of exogenous protein (44). Only solid surface-liquid biofilms were observed under our experimental conditions described here and no ripping or tearing of biofilm was observed.*

**5. Pg. 9, lines 201 & 203. 5’ and 3’ should be added to the primer sequences.**

Done
 **6. Pg. 10, line 234. Can ‘amounts’ be contextualised – were the biofilms thicker or more dense, or did they cover more of the microtitre well walls?**

This paragraph has been removed at the suggestion of Reviewer 1.
 **7. Pg. 10, line 238. Please indicate what type of biofilm was observed in these experiments. Is it possible that some wells produced A-L interface biofilms which were not registered by the crystal violet assay because the material was washed away?**

This has been addressed in the M&M.
 **8. Table 1. Could a contingency table / Chi Square analysis be conducted to see whether species abundance is dependent on abundance in river water and in biofilms?**

We wrestled with this but have no good solution using ChiSq to test individual populations. When testing the top 20 or top 50 populations, we can see no correlation between the biofilm community and the river community in a ChiSq test. Of course, the Anosim and Permanova tells us this as well. When we run pearson or spearman correlations there are only three populations in the top 25 pops of the 24 hr biofilm that correlated with their population size in the river. The caveat is that there is no indication of causality in this measure. We have added the following to the Table 1 section of the paper.

Only three populations of the top 25 in the 24-hour biofilm (*Flavobacterium* OUT00014, *Staphlococcus* OTU00017 and *Hydrogenophaga* OTU00003) had positive correlations with their population sizes in river water.

**9. Figure 2 does not specify what type of data are shown in the graph (means ± SE?).**

**The legend has been altered.**

***Fig 2. Resilience of single and double isolate biofilms to filtered and unfiltered river water as measured with crystal violet.*** *The plotted values are the average of four replicates and standard deviations are indicated with bars. Asterisks indicate responses that were statistically different (P value = 0.05 or less) from the cognate control 48-hour biofilm (black bars).*

**10. Figure 3 A & B do not show means and errors.**

Figure 3 has been recast, plotting means and standard deviation. The figure legend has been reworded.

**Fig 3. Resilience of Established Biofilm to Challenge with River Bacterial Populations as Determined by 16S Amplicon Sequencing.** Panel A, percent (average of four replicates) of the founding populations remaining in biofilms at 4, 8 and 24 hours. Panels B (F14 & F16 mixed biofilm) and C (F19 & F16 mixed biofilm), percent (average of four replicates) of the founding two-isolate biofilms remaining after 4, 8 and 24 hours. Percent is calculated based on the entire community (11,100 sequences), including isolates. Error bars display the standard deviation based on four replicates.

**11. Pg. 19, line 462. This statement is disingenuous especially after hydrophobicity is noted on the following page. Do the surfaces also differ in terms of microtopography which might aid recruitment from flowing water / protection from shear force, and flexibility which may also play a role in biofilm-formation and retention?**

Not exactly sure what the reviewer means. In line 462 we state that the only commonality between the egg and the microtiter plate is that both surfaces “are previously uncolonized”. On the following page we note that the microtiter surface is hydrophilic, not hydrophobic. There are differences in the “microtopography” in that the egg is highly cavitated. We have added a sentence about this.

*In addition, the egg’s surface is highly cavitated, providing many surface irregularities for bacteria to attach (42)* **12. Pg. 20, line 488. Can any comment be made of whether, in the microtitre wells used for the biofilm assays, there was any evidence of A-L biofilm formation, incomplete surface colonisation or possible ripping of biofilms from the surface (which may all result from aging biofilms and/or over vigorous washing and staining)?**

Addressed in the M&M.
 **13. Pg. 21, line 511. Some references should be provided to contextualise biofilm formation by P. aeruginosa strains isolated from fresh water and/or tested in fresh water-based medium.**

Addressed in the M&M as suggested.

*Pseudomonas aeruginosa* PAO1 was the generous gift of Dr. Michael Bagdasarian at Michigan State University. This strain was used as a strong positive control for biofilm formation and is routinely isolated from freshwater sources (44, 45, 46, 47) including the Red Cedar River (A. Chan & T.L. Marsh, unpublished), the source of river water for these studies.
