# Supplementary material for: The impact of primary colonizers on the community composition of river biofilm
Source: PLoS One. 2023 Nov 13;18(11):e0288040. doi: 10.1371/journal.pone.0288040 (PMC10642824; doi:10.1371/journal.pone.0288040)
Supplement: Attachment — Submitted filename: Response to Reviewers V2.docx [file pone.0288040.s007.docx]

PONE-D-22-31146R1

The impact of primary colonizers on the community composition of river biofilm

Angoshtari, Scribner & Marsh

PLOS ONE

**Response to reviewers, version 2.**

Below find our point-by-point response to the comments of our reviewers.

Best regards

Terence L. Marsh, Ph.D.

Assoc. Professor Emeritus

Dept. of Microbiology & Molecular Genetics

567 Wilson Road

Michigan State University

East Lansing, MI 48824

(517)-231-6033

MARSHT@msu.edu

**Reviewer #1**: The manuscript has improved, and I had a couple of follow ups that I think need to be addressed.

**Table 1:

I was referring to the lettering scheme with panels “A and B” and then the lowercase lettering scheme a, b, c, d, e being confusing and not intuitive. I still think it is. And the legend is not appropriate. There is no acknowledgement of panels A and B and a, d, and e are just results.

I don’t think it’s necessary to have it with A and B and then a. b. c. d. and e. My suggestion is to just refer to A and B only in the legend. Since everything else is labeled already describing what it is, then just include the other information in the legend without the reference to any letter. Basically, eliminate the a,b,c,d,e**

DONE. We have eliminated the small letters and have incorporated that information into the text.

**“Population” is also not the current term in the table or in the legend. These are OTUs representing unique taxa… But then you use phylotypes in some of the text but populations elsewhere. Is phylotype or OTU more appropriate here? Were you screening sequences again the database to generate phylotypes or clustering sequences for OTUs? This should be clear in the methods but it wasn't to me. Table 1 says OTUs. This is an inconsistency between population, OTU, and phylotypes is throughout the manuscript.**

Apologies for the confusion. We now note in the M&M that our definition of OTU is set at 97% similarity to 16S rRNA. Without question this statement should have been in the first version. With the OTU set at this traditional cutoff for species-level identifications, our view is that species = population = OTU = phylotype. However, to avoid confusion, we have eliminated the usage of “phylotype”.

**Phylotype changed to**…”population” in L261 L451 L457 L458 L563, and to “OTU “in L298 L300 L308 L439 L472 & L474 of Fig 6 legend L517 and eliminated from L455.

In addition, we have reduced the usage of “species” in the manuscript. The word “species” occurs twice in the introduction where we feel it is appropriate where referring in general terms to “aquatic species”. When we are referring to a “single-species biofilm”, we feel that the term “species” is appropriate and accurate inasmuch as we are referring to a biofilm derived from a pure culture, a species of bacteria (eg. L141). We have removed the term “species” when referring to “populations/OTUs” in the bacterial community of a river. Hence “a river community comprised of hundreds of species” has been changed to “a river bacterial community comprised of hundreds of populations”. We also cite references that use the phrase “species interactions” within a biofilm. We have retained this. In line 532 we refer to the stability of our single and double species biofilms as species specific. While we think this is an appropriate term, we have changed it to “isolate specific”. In the Discussion we use “species” when referring to “all free-living species”. The term “mixed species biofilms” is common in the literature and therefore the following phrase “through a temporal sequence of species additions” seems quite appropriate. Finally, in L599 we refer to a population/OTU of Desulfobacteriaceae as a species. We have changed this to population. We feel that population is an ecologically accurate term as we use it in this manuscript, referring to a collection of individual cells belonging to the same OTU-defined group or species. We use “population” when referring to any of the previously isolated strains used in our work. For example, in line 257 we refer to “the founding populations” of biofilm. However, immediately following this remark, we have changed “between the populations forming the river biofilm” to “between the detected OTUs forming the river biofilm” for clarity.

The following was added to the M&M.

OTU identification was at the 97% similarity level of 16S rRNA using the RDP Training set. This cutoff defines what we refer to as an “OTU” or “population”, used interchangeably herein.

Our usage of "OTU" is very limited. We used the term only 6 times in the text as indicated below (this refers to version 2 of the manuscript, the number goes up in version 3 due to replacement of all occurrences of “phylotype”). We first define OTU in the M&M. The second appearance is line 259 where we changed “population” to “OTU” in response to the reviewer. The third event is where we indicate that 10,000 OTUs were used for analysis in PAST. The remaining usage occurs in labels that are generated with MOTHUR as specific identifiers for OTUs (eg. *Staphlococcus* OTU00017, L310). We feel these identifiers are important to retain.

The following are where the term OTU has been retained as in the text of version 2.

L249 definition of OTU.

L253. Indicating that analysis in PAST was based on 10,000 OTUs as identified by analysis in MOTHUR.

L259. Changed “population” to “OTUs”

L310. Identification of specific OTUs identified by Mothur.

L469. Legend Fig 6. These are specific OTU identifiers from the Mothur analysis and are quite important to the analysis.

L521. This is another specific Mothur OTU identifier.

Again, we apologize for confusion regarding the terms OTU, population and species. We would prefer to be on the precise side of terminology but sometimes it is difficult. Two years ago, we submitted a paper on these same bacterial strains isolated from the sturgeon. We used the term “strain” throughout the paper when referencing the isolates. One reviewer argued that “strain” was inappropriate in this case because these isolates had not been “formally described”, even though we had previously presented full 16S rRNA sequence and cited information from full genome sequencing. We had to change all occurrences of “strain” to “isolate”. It’s not clear if the paper was improved.

**Were you screening sequences again the database to generate phylotypes or clustering sequences for OTUs?**

We find this sentence confusing. When running this type of community analysis, one is screening sequences against a database and binning sequences into OTUs. There is no either/or here, as we view it. We would certainly agree that the definition of species is elusive, controversial and a moving target. Is it 97% rRNA similarity or is it 99% or should it be an ANI value now that we are in the genome era? We have therefore removed the term “species” wherever there might be confusion when referencing bacterial populations within our studies, as described above.

RE: response from authors in R1:

"The cell concentration of the inoculum is irrelevant to the amount of biofilm formed in 48 hours over a wide range of initial cell concentrations. We have determined this in studies with 500-600 freshwater isolates, including those that we used in this study. "
…

"This issue is addressed above. Briefly, what is important here is the biofilm formed after 48 hours. Healthy 16-hour cultures were used as the inoculum. Each strain has its own unique
growth rate and ability to form biofilm. The biomass of biofilm produced by each strain was
strain-dependent. Under our experimental conditions the biofilm amounts as measured by crystal violet were consistent for each strain at 48 hours, over a broad range of inoculum concentration. "


**Okay, but I don’t see in the text or the snippet you added these specific comments that there was consistent biofilm with a range of inoculums. Is the evidence that inoculum size of differing ranges yields the same amount of biofilm at 48 hours something you can cite from previous work or is there data? Or personal comm?**

Added. Our extensive experience with the bacterial strains in this investigation indicated that overnight cultures (16 hrs) used as inocula, consistently produced strain-dependent amounts of biofilm at 24 and 48 hours. This was statistically confirmed in each experiment with four replicates for each treatment

**In general I find there is a lot figure and now table explanation in the text rather than just describing the text. Addressing this could make the results more concise.**

This is a difficult critique to address. “**…there is a lot of figure and now table explanation in the text…**”. We have tried to be as concise as possible while also making sure that the reader does not get overly distant from the data. I suspect that we have a different view of what the text accompanying the figures should be. **“…rather than just describing the text”** We are unsure what you mean by “describing the text”. We have gone through the results section several times now to reduce what we view as unnecessary verbiage. For example we have deleted the following including the following because it was repetitious (L332 of version2) In addition, *P. aeruginos*a PAO1, a strong biofilm-positive control, and *Serratia marcescens* RL10, a soil isolate that forms a considerably less robust biofilm compared to strain PAO1, were included. Of course, eliminating the table footers necessitated adding verbiage to the results section including the following. L302 version 3 - These account for 52% of all sequences. In Table 1, note that the numbers in parentheses following the taxonomic rank indicate the confidence level of phylogenetic assignment. L307 version 3 - The top 25 OTUs identified in 24-hour biofilm account for 84% of the community. We were surprised by this objection to the table footers inasmuch as this is a common feature of tables, including those published in PLOS ONE. We hope that the current version is satisfactory.

**Line 419 refers to supplemental material – is this suppose to be to a specific figure?**

Specific figure number has been added. “This separation could be seen in the early samples at 4 and 8 hours as well (S4 and S5 of supplemental material).”

**Number of references at line 579/580 seem a bit excessive.**

We have removed 15 references and retained 13 to cover 6 genera.

**Reviewer #2: Comments from the Reviewer of the Angoshtari et al. revised manuscript ‘The impact of primary colonizers on the community composition of river biofilm’ (PLoS ONE)

Angoshtari et al. present a revised manuscript which deals well with the comments I had made earlier. There are some very minor comments that should be addressed:

1. Line 50. It is arguable that all extant aquatic metazoans were present for millions of years; perhaps this dramatic statement can be rewritten as ‘Aquatic metazoans …’.**

This has been changed as suggested.

**2. Lines 56-57. This sentence seems repetitive and could be deleted.**
Deleted

**3. Line 101. Correct ‘I’.**
Corrected

**4. Pseudomonas aeruginosa PA01 was isolated from an Australian patient in 1954 and it is exceedingly unlikely that the exact same strain has been isolated since then – and certainly not from the Red Cedar River. The sentence in Lines 111 – 114 needs to be altered to say that ‘other strains of this species’ are routinely isolated from fresh water sources (the rest of the manuscript needs to be checked and corrected if necessary).**

Corrected. Thank you. Changed to “This strain was used as a strong positive control for biofilm formation and other strains of this species are routinely isolated from freshwater sources (44, 45, 46, 47) including the Red Cedar River (A. Chan & T.L. Marsh, unpublished), the source of river water for these studies.”

**5. Pseudomonas aeruginosa PA01 is referred to at various points in the manuscript but there is inconsistency in naming – a consistent approach should be adopted, e.g., Pseudomonas aeruginosa PA01 and thereafter P. aeruginosa PA01.**

DONE

**6. P. aeruginosa PA01 is not necessarily thought of as a model for river bacteria, and it should be made clear when it is first referred to (after the Methods) that P. aeruginosa strains are regularly recovered from freshwater systems.**

DONE. Added to L341; “While we have used *P. aeruginosa* PAO1 as a robust biofilm forming strain, as we point out in the Material and Methods section, strains of *P. aeruginosa* are routinely isolated from freshwater systems.”

**7. While a particular crystal violet assay was undertaken in this work, there are various forms of the assay, including washing steps and eluent. Perhaps referring to it as ‘the standard’ (Line 203 and possibly elsewhere) is inappropriate and it should be referred to as simply ‘the crystal violet assay’.**

L202, L335, L339, L558 have been changed. Line numbers are from version 2.

**8. Figure legends and possibly elsewhere. The term ‘average’ should be avoided as there are three possibilities; please replace with ‘mean’ throughout.**

L210, Fig 2 legend, Fig 3 legend, S2 Fig were changed.

**9. Line 340. The end of the sentence ‘in the biofilm that traps crystal violet’ is not needed and arguably, is incorrect: crystal violet binds to the bacterial peptidoglycan layer (and probably more generally to proteins as it stains fingers too).**

Deleted.

**10. Line 478. It is not necessary to specify ‘Earth’s’ microbiome unless there are substantive reports that microbial biomes in orbit don’t form biofilms.**

CHANGED TO “For example, we do not know if all free-living species of bacteria can form biofilms, but it has become apparent that a substantial fraction of bacteria in the environment exists as biofilm (67).” We have changed this but still feel that using the phrase “earth’s microbiome” does indicate the totality of bacteria on the planet and does not necessarily imply bugs anywhere else in the cosmos.

**11. Line 518. Delete ‘+’ as 2000 is the critical value discussed here.**

Done
